# Long-Term Evolution of SARS-CoV-2 in an Immunocompromised Patient with Non-Hodgkin Lymphoma

**DOI:** 10.1128/mSphere.00244-21

**Published:** 2021-07-28

**Authors:** Vítor Borges, Joana Isidro, Mário Cunha, Daniela Cochicho, Luís Martins, Luís Banha, Margarida Figueiredo, Leonor Rebelo, Maria Céu Trindade, Sílvia Duarte, Luís Vieira, Maria João Alves, Inês Costa, Raquel Guiomar, Madalena Santos, Rita Cortê-Real, André Dias, Diana Póvoas, João Cabo, Carlos Figueiredo, Maria José Manata, Fernando Maltez, Maria Gomes da Silva, João Paulo Gomes

**Affiliations:** a Bioinformatics Unit, Department of Infectious Diseases, National Institute of Health Dr. Ricardo Jorge (INSA), Lisbon, Portugal; b Clinical Pathology–Virology Lab, Instituto Português de Oncologia de Lisboa, Lisbon, Portugal; c Serviço de Hematologia, Instituto Português de Oncologia de Lisboa Francisco Gentil, Lisbon, Portugal; d Innovation and Technology Unit, Department of Human Genetics, National Institute of Health Dr. Ricardo Jorge (INSA), Lisbon, Portugal; e Centre for Vectors and Infectious Diseases Research, Department of Infectious Diseases, National Institute of Health Dr. Ricardo Jorge (INSA), Lisbon, Portugal; f National Reference Laboratory for Influenza and other Respiratory Viruses, Department of Infectious Diseases, National Institute of Health Doutor Ricardo Jorge (INSA), Lisbon, Portugal; g Laboratório de Biologia Molecular, Serviço de Patologia Clínica do CHULC, Lisbon, Portugal; h Serviço de Doenças Infecciosas do Hospital de Curry Cabral-CHULC, Lisbon, Portugal; i Serviço de Hematologia, Instituto Português de Oncologia de Lisboa Francisco Gentil, Lisbon, Portugal; University of Pittsburgh School of Medicine

**Keywords:** SARS-CoV-2, long-term infection, convergent evolution, immunocompromised host, genome sequencing

## Abstract

Recent studies have shown that persistent SARS-CoV-2 infections in immunocompromised patients can trigger the accumulation of an unusual high number of mutations with potential relevance at both biological and epidemiological levels. Here, we report a case of an immunocompromised patient (non-Hodgkin lymphoma patient under immunosuppressive therapy) with a persistent SARS-CoV-2 infection (marked by intermittent positivity) over at least 6 months. Viral genome sequencing was performed at days 1, 164, and 171 to evaluate SARS-CoV-2 evolution. Among the 15 single-nucleotide polymorphisms (SNPs) (11 leading to amino acid alterations) and 3 deletions accumulated during this long-term infection, four amino acid changes (V3G, S50L, N87S, and A222V) and two deletions (18-30del and 141-144del) occurred in the virus Spike protein. Although no convalescent plasma therapy was administered, some of the detected mutations have been independently reported in other chronically infected individuals, which supports a scenario of convergent adaptive evolution. This study shows that it is of the utmost relevance to monitor the SARS-CoV-2 evolution in immunocompromised individuals, not only to identify novel potentially adaptive mutations, but also to mitigate the risk of introducing “hyper-evolved” variants in the community.

**IMPORTANCE** Tracking the within-patient evolution of SARS-CoV-2 is key to understanding how this pandemic virus shapes its genome toward immune evasion and survival. In the present study, by monitoring a long-term COVID-19 immunocompromised patient, we observed the concurrent emergence of mutations potentially associated with immune evasion and/or enhanced transmission, mostly targeting the SARS-CoV-2 key host-interacting protein and antigen. These findings show that the frequent oscillation in the immune status in immunocompromised individuals can trigger an accelerated virus evolution, thus consolidating this study model as an accelerated pathway to better understand SARS-CoV-2 adaptive traits and anticipate the emergence of variants of concern.

## INTRODUCTION

Long-term persistence of SARS-CoV-2 in immunocompromised patients has been reported (1–4). These infections are usually characterized by intermittently detectable SARS-CoV-2 RNA for several months and a within-patient virus evolutionary trajectory marked by accumulation of an unusually high number of mutations (1–4). Immunocompromised patients are sometimes treated with convalescent plasma and with the antiviral drug remdesivir, which can trigger viral population shifts, shaping the dynamics of SARS-CoV-2 evolution (1–4). The first report of persistence and evolution of SARS-CoV-2 in an immunocompromised patient, by Choi and colleagues (1), not only unveiled a scenario of accelerated viral evolution, but also showed that infectious virus can be recovered from nasopharyngeal samples for several months. In another study, Kemp et al. (2) reported a fatal SARS-CoV-2 escape from neutralizing antibodies in an immune suppressed patient treated with convalescent plasma. SARS-CoV-2 immune evasion could be linked to the emergence of a dominant viral strain bearing mutations in the key antigen (spike protein), potentially altering the recognition by antibodies and thus the sensitivity to convalescent plasma (2). These and other studies monitoring persistent infections in immunocompromised patients (1–4) have also highlighted the emergence of identical mutations in independent patients, supporting that persistent RNA positivity can drive convergent adaptive evolution of SARS-CoV-2. Corroborating the potential biological relevance of those recurrent mutations, some of them are predicted to affect SARS-CoV-2 affinity to ACE-2 receptors (e.g., Y453F), to be potentially involved in immune evasion (e.g., E484K), or to increase entry efficiency (e.g., ΔH69/ΔV70) (2, 5–8). Of note, the emergent and highly concerning SARS-CoV-2 lineage B.1.1.7 (VOC 202012/01) (9), likely originated in the United Kingdom, was hypothesized to be a result of virus evolution in a chronically infected individual (index patient), as revealed by its unusually high genetic divergence (with key amino acid changes predominantly affecting the spike protein) (9).

Here, we report a case of an immunocompromised patient (non-Hodgkin lymphoma patient under immunosuppressive therapy) with SARS-CoV-2 positivity spanning over at least 196 days. Although no convalescent plasma therapy was administered, the long-term within-patient evolution of SARS-CoV-2 was still marked by a mutation accumulation signature that not only resembles other similar reports, but also highlights novel potentially biologically relevant mutations.

## RESULTS

### Clinical case.

A female patient, age 61, diagnosed with stage IVB non-Hodgkin diffuse large B-cell lymphoma, was admitted to hospital A on 10 June 2020, due to a bacterial infection in the setting of postchemotherapy neutropenia, testing negative for SARS-CoV-2 at admission (Fig. S1). After the detection of a cluster of SARS-CoV-2-positive cases in that hospital, the patient was again screened for SARS-CoV-2 6 days later, testing positive, while being asymptomatic. She was transferred to a COVID-19 ward of hospital B, where she stayed for 57 days. During this period, she evolved from mildly symptomatic to respiratory insufficiency requiring invasive mechanical ventilation, from which she recovered slowly. She was treated with remdesivir for 10 days and high-dose corticosteroids for 7 days during the hospitalization period, being discharged on day 58. During the next months, she remained intermittently symptomatic, ranging from fatigue, cough, and low-grade fever to shortness of breath requiring supplemental oxygen at home. Residual pneumonitis with extensive fibrotic changes remained evident on computed tomography scan. Meningeal lymphoma progression required weekly intrathecal chemotherapy in October and systemic methotrexate administration on day 143, after which systemic symptoms, cough, and dyspnea became more evident. Aiming at further investigation of these symptoms, the patient was readmitted on day 163 to a COVID ward. Bronchoalveolar lavage and transbronchial biopsy were performed, and other possible causes were excluded (other respiratory viruses, bacterial causes of atypical pneumonia, Pneumocystis jirovecii pneumonia, noninfectious causes of intersticial pneumonitis/fibrosis). No empirical antibiotic treatment was administered. Exertional dyspnea requiring supplemental oxygen remained the most prominent clinical feature at discharge. After discharge, intermittent symptoms and partial respiratory insufficiency persisted. Reappearance of neurological symptoms led again to intrathecal chemotherapy, with partial relief. SARS-CoV-2 reverse transcriptase PCR (RT-PCR) positivity was intermittent during the 197-day long-term infection (details in Table S1 and Fig. S1). The patient was negative for the antibodies anti-SARS-CoV-2 and IgG/IgM (day 171).

10.1128/mSphere.00244-21.1 TABLE S1 Description of clinical samples subjected to SARS-CoV-2 RT-PCR test. Download Table S1, PDF file, 0.01 MB. Copyright © 2021 Borges et al. 2021 Borges et al. https://creativecommons.org/licenses/by/4.0/ This content is distributed under the terms of the Creative Commons Attribution 4.0 International license.

10.1128/mSphere.00244-21.3 FIG S1 Timeline of key clinical events during the long-term SARS-CoV-2 infection of an immunocompromised patient with non-Hodgkin lymphoma. Download FIG S1, PDF file, 0.6 MB. Copyright © 2021 Borges et al. 2021 Borges et al. https://creativecommons.org/licenses/by/4.0/ This content is distributed under the terms of the Creative Commons Attribution 4.0 International license.

### Genomic investigation.

**FIG 1:**
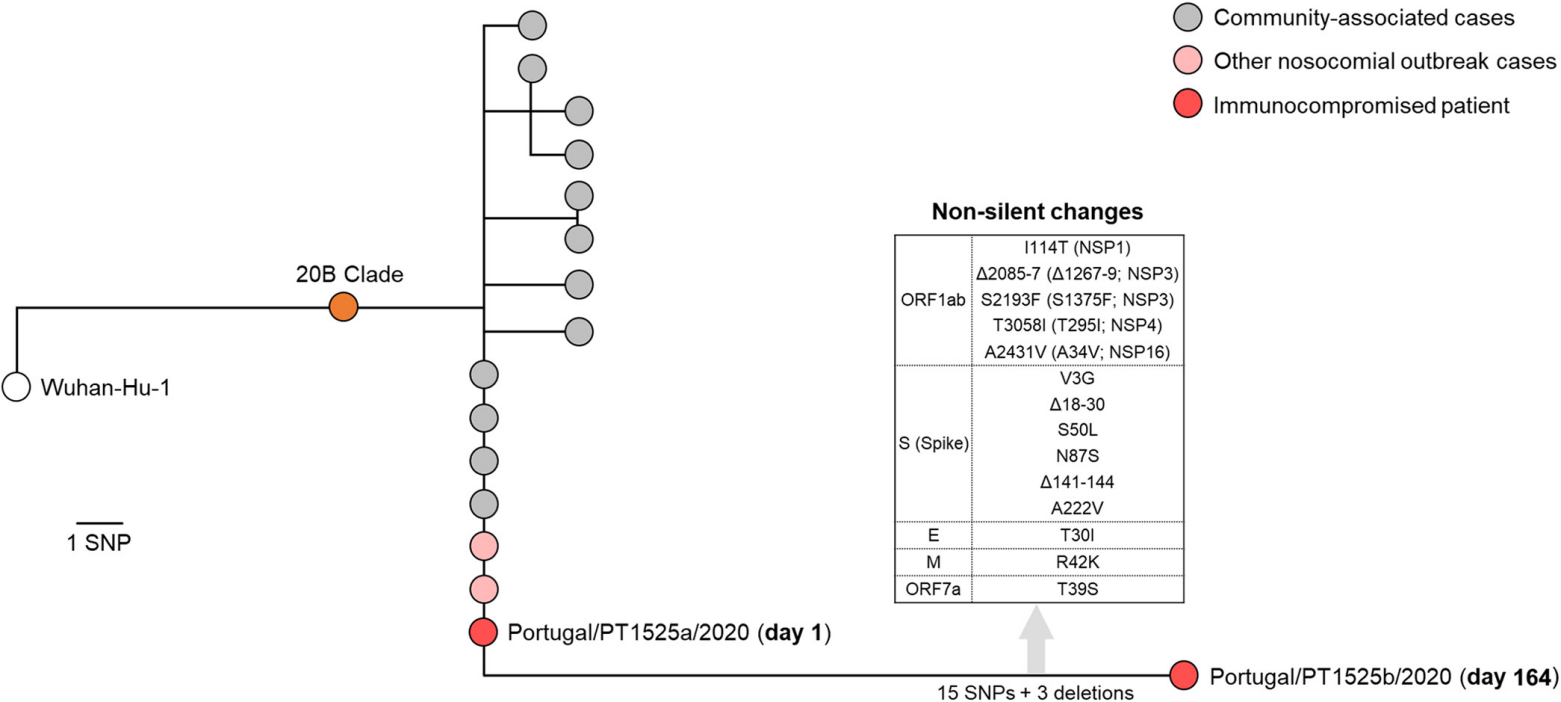
Integration of the viral genome sequences recovered during the long-term SARS-CoV-2 infection of an immunocompromised individual in the phylogenetic diversity of SARS-CoV-2 in Portugal (https://insaflu.insa.pt/covid19/). The immunocompromised patient mostly likely acquired the infection in the context of the nosocomial outbreak detected in hospital A by mid- to late June, as revealed by the detection on day 1 of the same genetic profile observed in other outbreak-associated inpatients of the same hospital. During 164 days of infection, SARS-CoV-2 accumulated 15 SNPs and 3 deletions (Table 1), including several nonsilent mutations in the spike coding gene.

In order to confirm the long-term COVID-19 infection (and exclude the reinfection hypothesis) and monitor SARS-CoV-2 within-patient evolution, viral genome sequencing was performed, as previously described (10), on a nasopharyngeal swab obtained on day 1 and on sputum and bronchoalveolar lavage specimens collected on days 164 and 171, respectively (Tables S1 and 2). Although viral culture using the clinical specimen collected on day 164 was also attempted, no virus recovery was achieved. Integration of the viral genome sequence obtained on day 1 (Portugal/PT1525a/2020; GISAID accession number EPI_ISL_941339) in the phylogenetic diversity of SARS-CoV-2 in Portugal (https://insaflu.insa.pt/covid19/) confirmed that the immunocompromised patient most likely acquired the infection in the context of the nosocomial outbreak detected in hospital A by mid- to late June. In fact, the genome sequence was found to be identical to that of other outbreak-associated inpatients in the same hospital, falling within a cluster also enrolling COVID-19 cases detected at community level (https://insaflu.insa.pt/covid19/) (Fig. 1). The outbreak-causing SARS-CoV-2 belongs to COG-UK lineage B.1.1.401 and Nextstrain clade 20B, carrying the spike amino acid change D614G (Table 1). It diverges from the Wuhan-Hu-1/2019 reference genome (GenBank accession number MN908947.3) (11) and the clade 20B root by 10 and 3 single-nucleotide polymorphisms (SNPs), respectively (Table 1). Analysis of the SARS-CoV-2 genome sequence collected at day 164 (Portugal/PT1525b/2020; GISAID accession number EPI_ISL_941340) confirmed the exact ancestral genomic backbone of the virus collected at day 1, with the notable addition of 3 deletions and 15 SNPs (Table 1). Of note, two SNPs (T21570G and C21771T, leading to spike amino acid changes V3G and S50L) detected in this sample displayed intrahost intermediate frequency (52% and 93%), suggesting that they might be recent emerging mutations. A partial genome sequence was obtained on day 171, revealing no additional mutations. Among the extra 15 SNPs detected in the evolved SARS-CoV-2, 11 lead to amino acid alterations and the remaining four are silent. In total, four amino acid changes (V3G, S50L, N87S, and A222V) and two deletions (18-30del and 141-144del) occurred in the virus spike protein. Remarkably, as detailed in Table 1, several mutations detected during the long-term infection monitored in this study have been independently observed in similar studies focusing on SARS-CoV-2 evolution in chronically infected individuals.

**TABLE 1:** List of mutations accumulated during the long-term SARS-CoV-2 infection of an immunocompromised patient^*a*^

Genome position^*b*^	Nucleotide profile			Target of mutation	Amino acid change (alternative annotation)	Similar report(s)
	Wuhan-Hu-1 (GenBank accession no. MN908947)	Sample 1 (day 1)	Sample 11 (day 164)			
241*	C	**T**	**T**	5′ UTR		
**606**	T	T	**C**	**ORF1ab**	**I114T (NSP1)**	
3037*	C	**T**	**T**		Silent	
**4832**	C	C	**T**		**Silent**	
**5020**	C	C	**T**		**Silent**	
**6518–6526**	No deletion	No deletion	**9-bp deletion**		**Δ2085-7 (KIT) (Δ1267-9; NSP3)**	
**6843**	C	C	**T**		**S2193F (S1375F; NSP3)**	
7119	C	**T**	**T**		S2285F	
**9438**	C	C	**T**		**T3058I (T295I; NSP4)**	4
14408*	C	**T**	**T**		P314L (NSP12b)	
**20759**	C	C	**T**		**A2431V (A34V; NSP16)**	
**21570**	T	T	**G**	**S (Spike)**	**V3G**	
**21610–21648**	No deletion	No deletion	**39-bp deletion**		**Δ 18-30**	1 ^*c*^
**21711**	C	C	**T**		**S50L**	4
**21822**	A	A	**G**		**N87S**	
**21983–21994**	No deletion	No deletion	**12-bp deletion**		**Δ 141-144 (LGVY)**	1, 3, 4, 5^*c*^
**22227**	C	**C**	**T**		**A222V**	
**23290**	C	C	**T**		**silent**	
23403*	A	**G**	**G**		D614G	
**24829**	T	T	**C**		**Silent**	
25677	G	**T**	**T**	**ORF3a**	L95F	
26019	A	**C**	**C**		Silent	
**26333**	C	C	**T**	**E**	**T30I**	
**26647**	G	G	**A**	**M**	**R42K**	
**27508**	A	A	**T**	**ORF7a**	**T39S**	
28881–28883*	GGG	**AAC**	**AAC**	**N**	203-204 (RG > KR)	

a Nucleotide/amino acids in bold with white background reflect mutations compared with the Wuhan-Hu-1 reference sequence. Nucleotide/amino acids in bold with gray background indicate mutations accumulated during the long-term infection. None of the emerging mutations was found as a “minor variant” mutation in the day 1 sample. Also, only two mutations (T21570G and C21771T) presented less than 95% intrapatient frequency at day 164.

b Genome positions refer to the reference SARS-CoV-2 Wuhan-Hu-1/2019 sequence (GenBank accession number MN908947). Nextstrain 20B clade markers are labeled with an asterisk (*).

c The deletion partially overlaps with other deletions reported.

## DISCUSSION

In the present study, we report the long-term evolution of SARS-CoV-2 in an immunocompromised patient with non-Hodgkin lymphoma. In line with previous findings (1–4), SARS-CoV-2 underwent accelerated and potentially adaptive evolution within the host, with 18 changes being accumulated after 164 days. This corresponds to a rate of 1.3 × 10^−3^ mutations per site per year (i.e., ∼40 mutations per genome per year), which exceeds the estimated average rate of evolution of SARS-CoV-2 (around 8 × 10^−4^ mutations per site per year, i.e., around 23 mutations per genome per year) (12). In particular, SARS-CoV-2 evolved some of the exact same mutations seen in other immunocompromised individuals, most of them altering spike, the key host-interacting protein and antigen. The first example is the spike 141 to 144 deletion (amino acids LGVY). Indeed, this deletion also emerged during SARS-CoV-2 evolution in another immunocompromised patient with non-Hodgkin lymphoma (detection after 132 days) (4), in a patient with severe antiphospholipid syndrome (detection at day 152) (1), and in an asymptomatic immunocompromised patient with cancer (detection after 70 days) (3). Of note, this recurrent observation of spike 141 to 144 deletion in chronically infected individuals corroborates the highly plausible hypothesis that the emergent SARS-CoV-2 lineage B.1.1.7, which harbors the spike Y144 deletion, resulted from virus evolution in a chronically infected individual (5). Concordantly, identical or similar recurrent deletions that alter position 144 and adjacent positions have been shown to alter SARS-CoV-2 antigenicity, most likely driving adaptive evolution (13, 14). Another relevant recurrent mutation detected in the present study is the spike S50L amino acid change, as it was also detected during prolonged COVID-19 in another lymphoma patient (4). Although no experimental data are available about the functional role of S50L (which falls within the spike N-terminal domain, NTD), recent computational analysis suggests that it might have strong stabilizing effects on SARS-Cov-2 full-length spike protein (15). In the present study, we detected another large deletion in spike, leading to the loss of amino acids 18 to 30 (LTTRTQLPPAYTN). This region within the NTD might be of particular functional interest, as another large deletion in the proximal protein region (amino acids 12 to 18) was found to emerge 142 days after SARS-CoV-2 evolution in an immunocompromised patient (1). Notably, a spike mutation (L18F) affecting this region has shown strong signs of convergent evolution, being harbored by the variant of concern (VOC) P.1 and being present in a high proportion of VOC B.1.351 viruses (16). Outside spike, we highlight the SNP C9438T leading to a T295I amino acid change in NSP4 protein, as this exact mutation was detected during persistence and evolution of SARS-CoV-2 in another patient with non-Hodgkin diffuse B-cell lymphoma (4). NSP4 protein is expected to be involved in membrane rearrangements that are crucial for viral propagation (17), so this recurrent emergence in lymphoma patients is intriguing and warrants investigation.

Although the spike A222V mutation (within the NTD) has not yet been reported to recurrently emerge during long-term infections, it has been specially focused by the scientific community. In fact, it is shared by a SARS-CoV-2 lineage (B.1.177; Nextstrain clade 20A.EU1), likely emerging in Spain by summer 2020, that markedly increased in frequency worldwide (18). In addition, another spike A222V-bearing variant was the cause of one of the first reinfection cases reported worldwide (19). A222V is also predicted to be located within one of the epitopes recognized by unexposed humans (20). Altogether, our results consolidate the expectation that A222V might have a still to be disclosed functional impact on spike host-interacting activities.

Intriguingly, our report describes accelerated SARS-CoV-2 within-patient evolution in the absence of convalescent plasma therapy. While the lack of plasma-induced selective pressure might explain the lack of mutations affecting the epitope-enriched spike receptor binding domain (RBD), a rapid viral evolution targeting other key spike regions was still triggered. Considering that this non-Hodgkin lymphoma patient was under treatment with anti-CD20 antibodies before infection and was subjected to intrathecal and systemic chemotherapy during the course of the SARS-CoV-2 infection, it is very likely that the patient was particularly immunosuppressed, thus promoting viral adaptation. In fact, our study highlights novel potentially biologically relevant mutations, while it consolidates a scenario of SARS-CoV-2 convergent evolution in immunocompromised individuals, which is a hallmark of adaptive evolution. It also reinforces the need to monitor the virus evolution in immunocompromised individuals, not only to identify novel adaptive traits of SARS-CoV-2, but also to mitigate the risk of introducing “hyper-evolved” adapted variants in the community (in the studied case, no secondary cases were identified and no similar mutation profile was reported in the GISAID database, so onward transmission of the evolved variant seems unlikely). For example, the evolution toward alteration of epitopes might have an unpredictable impact on vaccine efficacy. Ultimately, this study highlights the need to revisit the approaches for the follow-up of COVID-19 immunocompromised patients, namely, the current recommendations for isolation and personal protection after discharge.

### Ethical declaration.

Verbal and written informed consents were obtained from the patient to allow the use of the clinical and virological data during prolonged infection. The SARS-CoV-2 genome sequencing study was approved by the Ethical Committee (“Comissão de Ética para a Saúde”) of the Portuguese National Institute of Health.

## MATERIALS AND METHODS

### Clinical specimens and RT-PCR testing.

Multiple respiratory clinical specimens were collected from the immunocompromised patient for SARS-CoV-2 RT-PCR testing during the study period (Table S1). Hospital A applied the RT-PCR test Seegene Allplex SARS COV 2 assay (until 30 June, v1—gene E, RdRP, and N; after 1 July, v2—gene E, RdRP/S, and N, analytical sensibility of 50 RNA copies/PCR), while hospital B used for nasopharyngeal/oropharyngeal specimens the rRT- PCR test Cobas SARS-CoV-2, analytical sensibility of 32 copies/ml (21 to 73 copies/ml; confidence interval [CI] 95%) (gene E) and 25 copies/ml (17 to 58 copies/ml; CI 95%) (gene ORF1ab) and for sputum, the novel coronavirus (2019-nCoV) RT-PCR detection assay (Fosun 2019-nCov qPCR), analytical sensibility of 300 copies/ml (gene E, ORF1ab and N). SARS-CoV-2-positive RNA samples were sent to the National Institute of Health (INSA), Ricardo Jorge, for SARS-CoV-2 whole-genome sequencing and bioinformatics analysis.

### SARS-CoV-2 genome sequencing.

Genome sequencing was performed at INSA following an amplicon-based whole-genome amplification strategy using tiled, multiplexed primers (21), according to the ARTIC network protocol (https://artic.network/ncov-2019; https://www.protocols.io/view/ncov-2019-sequencing-protocol-bbmuik6w) with slight modifications, as previously described (10). In brief, after cDNA synthesis, whole-genome amplification was performed using two separate pools of tiling primers (pools 1 and 2; primers version 3 [218 primers] were used for all samples: https://github.com/artic-network/artic-ncov2019/tree/master/primer_schemes/nCoV-2019). The two pools of multiplexed amplicons were then pooled for each sample, followed by post-PCR cleanup and Nextera XT dual-indexed library preparation, according to the manufacturers’ instructions. Sequencing libraries were paired-end sequenced (2 × 150 bp) on an Illumina NextSeq 550 apparatus, as previously described (10). All bioinformatics analysis (from read quality control to variant detection/inspection, sequence consensus generation, and minor variant analysis) was conducted using the online (and locally installable) INSaFLU platform (https://insaflu.insa.pt/) (22), as previously described (10). The genome sequence of SARS-CoV-2 Wuhan-Hu-1/2019 virus (GenBank accession number MN908947) was used as reference for mapping and single nucleotide variant (SNV) annotation (11). Regions with a depth of coverage below 10-fold were automatically masked in the INSaFLU pipeline by placing undefined bases “N” in the consensus sequence. Low-coverage regions were visually inspected on “.bam” files using Integrative Genomics Viewer (IGV), and the error-prone position 1871 was excluded. SNVs were assumed in consensus when they displayed more than 50% intrapatient frequency. Coronapp (http://giorgilab.dyndns.org/coronapp/) (23) was applied to refine the impact of mutations at the protein level. Clade and lineage assignments were performed using Nextclade (https://clades.nextstrain.org/) and Phylogenetic Assignment of Named Global Outbreak Lineages (Pangolin) (https://pangolin.cog-uk.io/) (24), respectively.

### Cell culture.

The SARS-COV-2 virus isolation attempt was performed in a biosafety level 3 (BSL3) laboratory at INSA. A clinical specimen (sputum, collected on day 164) was used for infecting Vero E6 cells, which were maintained in Eagle’s minimum essential medium (MEM; Gibco, UK) supplemented with 10% fetal bovine serum, penicillin (0,6μg/ml), and streptomycin (60 μg/ml). The sample was diluted in MEM (2×, 4×, and 8×), and 100 μl of each dilution was inoculated onto 25-cm^3^ flasks with a 70% monolayer of cells prepared 24 h before and washed with phosphate-buffered saline (PBS). The inoculated cells were incubated for 1 h at 37°C, 5% CO_2_, to allow virus adsorption. After that, 10 ml of MEM was added to each flask. The cultures were incubated at 37°C, 5% CO_2_, and observed daily for cytopathic effect (CPE). After 3 days, none of dilutions showed CPE. Despite the negative result, a blind passage was made, and new cells were infected by repeating the first passage method. Again, after 3 days, none of dilutions at the new passage showed CPE.

### SARS-CoV-2 serology.

*In vitro* qualitative detection of antibodies to SARS-CoV-2 in human serum was performed using Roche Elecsys anti-SARS-CoV-2 assay. This assay measures total immunoglobulins directed toward a recombinant nucleocapsid protein from SARS-CoV-2, reporting a ratio of specimen electrochemiluminescent signal to calibrator.

### Data availability.

The SARS-CoV-2 genome sequences generated in this study were uploaded to the GISAID database (https://www.gisaid.org/). The accession numbers are provided in Table S2. Integration of the sequence data generated in this study on behalf of the SARS-CoV-2 genetic diversity in Portugal can be consulted at https://insaflu.insa.pt/covid19/.

10.1128/mSphere.00244-21.2 TABLE S2 Sequencing data of SARS-CoV-2 genome consensus sequences obtained at day 1 and day 164. Download Table S2, PDF file, 0.2 MB. Copyright © 2021 Borges et al. 2021 Borges et al. https://creativecommons.org/licenses/by/4.0/ This content is distributed under the terms of the Creative Commons Attribution 4.0 International license.
