# Supplementary material for: Long-Term Evolution of SARS-CoV-2 in an Immunocompromised Patient with Non-Hodgkin Lymphoma
Source: mSphere. 2021 Jul 28;6(4):e00244-21. doi: 10.1128/mSphere.00244-21 (PMC8386466; doi:10.1128/mSphere.00244-21)
Supplement: TABLE S1 [file msphere.00244-21-st001.pdf]

Supplementary Material

Supplementary Table 1. Description of clinical samples subjected to SARS-CoV-2 RT-PCR test.

Sample number	Date of collection	Clinical specimen	**RT-PCR result** (Cycle threshold obtained for each gene target)	Hospital
**0**	2020-06-10 (**day 0**)	NP/OP	Negative	A
**1***	2020-06-16 (**day 1)**	NP/OP	**Positive** (E: 20.48; RdRP: 21.57; N: 22.22)	A
**2**	2020-06-30 (**day 15**)	NP/OP	**Positive** (E- detected; ORF 1ab-detected)	B
**3**	2020-07-07 (**day 22**)	NP/OP	**Positive** (E- detected; ORF 1ab-detected)	B
**4**	2020-08-11 (**day 57**)	NP/OP	**Positive** (E: 32.8; ORF 1ab-not detected; N:31.8)	B
**5**	2020-09-03 (**day 80**)	NP/OP	**Positive** (E: 38.20; RdRP/S: 38.98, N: 37.55)	A
**6**	2020-09-21 (**day 98**)	NP/OP	Negative	A
**7**	2020-11-03 (**day 141**)	NP/OP	Negative	A
**8**	2020-11-07 (**day 145**)	NP/OP	Negative	A
**9**	2020-11-24 (**day 162**)	NP/OP	**Positive** (E: 36.52; RdRP/S: 38.58, N: 36.45)	A
**10***	2020-11-26 (**day 164**)	S	**Positive** (E: 21.97; ORF1ab: 22.53, N: 21.29)	B
**11***	2020-12-03 (**day 171**)	BAL	**Positive** (E: 28.23; ORF1ab: 27.83, N: 26.64)	INSA
**12**	2020-12-29 (**day 197**)	NP/OP	**Positive** (E: 35; RdRP/S: 34.63; N: 34.70)	A

* Samples subjected to SARS-CoV-2 genome sequencing; OP: oropharyngeal swab; NP: nasopharyngeal swab; S: sputum; BAL: Bronchoalveolar lavage
