# Supplementary material for: Long-Term Evolution of SARS-CoV-2 in an Immunocompromised Patient with Non-Hodgkin Lymphoma
Source: mSphere. 2021 Jul 28;6(4):e00244-21. doi: 10.1128/mSphere.00244-21 (PMC8386466; doi:10.1128/mSphere.00244-21)

Supplementary Material

Supplementary Figure 1. Timeline of key clinical events during the long-term SARS-CoV-2 infection of an immunocompromised patient with non-Hodgkin lymphoma.

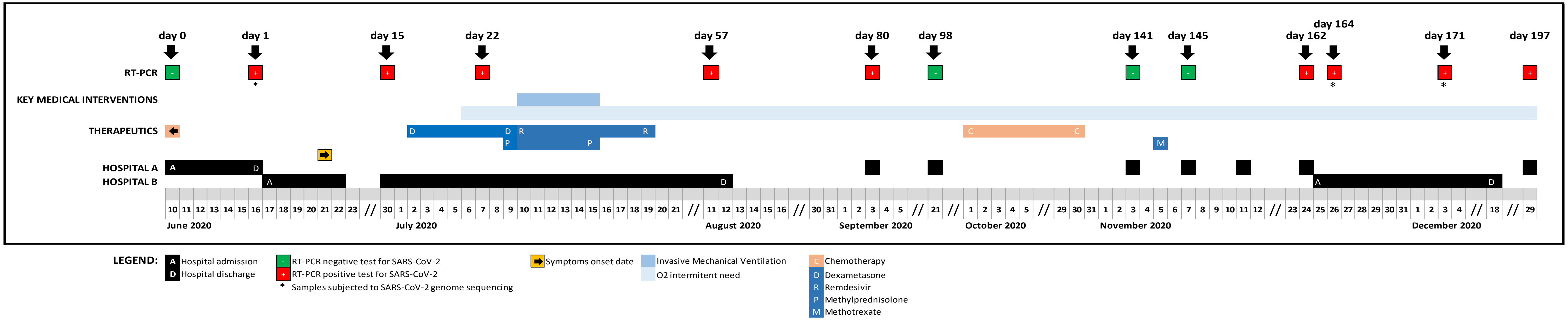

Supplement: FIG S1 [file msphere.00244-21-sf001.pdf]
