# Supplementary material for: Long-Term Evolution of SARS-CoV-2 in an Immunocompromised Patient with Non-Hodgkin Lymphoma
Source: mSphere. 2021 Jul 28;6(4):e00244-21. doi: 10.1128/mSphere.00244-21 (PMC8386466; doi:10.1128/mSphere.00244-21)
Supplement: TABLE S2 [file msphere.00244-21-st002.pdf]

Supplementary Material

Supplementary Table 2. Sequencing data of SARS-CoV-2 genome consensus sequences obtained at day 1 and day 164.

Sequence ID	Date of collection	Mean coverage	% of genome with ≥1-fold	% of genome with ≥10-fold	GISAID accession
Portugal/PT1525a/2020	2020-06-16 (**day 1)**	3855x	99,8%	99,7%	EPI_ISL_941339
Portugal/PT1525b/2020*	2020-11-26 (**day 164**)	2297x	99,6%	97,7%	EPI_ISL_941340

* Regions with depth of coverage below 10-fold were automatically masked in INSaFLU pipeline by placing undefined bases "N" in the consensus sequence. One of these small regions (22986-23122) in the Portugal/PT1525b/2020 sequence falls within S gene (coverage between 2- and 9-fold). Due to its biological relevance, all reads were inspected, showing no differences for the reference genome (this region was then unmasked).
